# 

**Published:** 1920-09-11

**Authors:** 


### Matrons' and Sisters' Appointments.

AXBRIDGE UNION INFIRMARY, SOMERSET.—Miss Maria Lati has been appointed head nurse. She was trained at the Middlesex and Queen Charlotte's Hospitals, and has since nursed in various Australian hospitals.

CITY OF CARLISLE MATERNITY HOSPITAL.—Miss F. Drewitt has been appointed matron. She was trained at the Royal Free Hospital and at the Rotunda Hospital, Dublin, and has since been ward and theatre sister at the Royal Free Hospital.

THE GUEST HOSPITAL AND EYE INFIRMARY, DUDLEY.—Miss Dorothea Watson has been appointed matron. She was trained at the Royal Infirmary, Leicester, where she was later ward sister. Miss Watson has also been ward sister at the Norfolk and Norwich Hospital, assistant matron at the City of London Hospital for Diseases of the Chest and at the Bradford Royal Infirmary, and matron of the General Hospital, Newark-on-Trent.

HARTON HOSPITAL, SOUTH SHIELDS.—Miss Effie Oram has been appointed head night sister. She was trained at the Stoke-on-Trent Union Hospital, and was later on the staff of Selly Oak Hospital, Birmingham.

ISLE OF THANET UNION, HILL HOUSE, MINSTER.—Miss Ethel Clarke has been appointed superintendent nurse. She was trained at Bagthorpe Infirmary, Nottingham, and has held various nursing appointments.

LAMBETH PARISH CHILDREN'S INFIRMARY, WEST NORWOOD.—Miss Gertrude Margaret Aubyn has been appointed sister-in-charge. She was trained at Kensington Infirmary, has been sister under the Kensington Board, school nurse under the Swindon Education Committee, and sister at Bradfield Infirmary.

MATERNITY AND CHILD WELFARE HOME, EASTBOURNE.—Miss Anna I. Dumbell has been appointed sister. She was trained at Bootle Borough Hospital, Liverpool, and received her midwifery training at the Elizabeth Garrett Anderson Hospital. Miss Dumbell has since been sister and matron of the Leaf Hospital, Eastbourne, and sister at the Liverpool Skin Hospital.

STOKE-UPON-TRENT UNION HOSPITAL, NEAR NEWCASTLE, STAFFS.—Miss Z. Meal and Miss Annie Whiteley have been appointed ward sister and sister respectively. They were both trained at Halifax Poor-Law Hospital, and received their midwifery training at Liverpool Maternity Hospital, and have both done private nursing at Hull.

TUBERCULOSIS HOSPITAL, NORTHAMPTON.—Miss Lucy M. Roberts has been apointed sister. She was trained at Bootle Borough Hospital.

### Wills of Medical Men.

MR. ARTHUR BLOUNT, L.R.C.P., M.R.C.S., L.S.A., of Lawrence Road, Hove, formerly of Alexandra Road, Hornsey, has left estate of the gross value of £10,544.

MR. WHEELTON HIND, M.B., of Roxeth House, Stoke-on-Trent, left estate to the value of £43,243.

DR. D. J. WILLIAMS, of Greenfield House, Llanelly, Carmarthen, one of the oldest medical practitioners in the town, left estate to the value of £27,489.

DR. D. E. BERNARD, of Sydenham Road, Cotham, Bristol, late assistant surgeon to Bristol Royal Infirmary, left estate to the value of £8,981.

DR. PHILIP RHYS GRIFFITHS, of Newport Road, Cardiff, late senior surgeon of King Edward VII. Hospital, Cardiff, and lieutenant-colonel in the R.A.M.C., left estate valued at £6,813.

### Gifts and Bequests.

VICTORIA HOSPITAL, Accrington, has been left £1,000 under the will of the late Sir George McAlpine.

SIR GEORGE WATSON MCALPINE, of Accrington, Lancs, has left £1,000 to the Victoria Hospital, Accrington.

A LEGACY of £250 for the better equipment of the operating-theatre has been left to Llanelly Hospital by Dr. David James Williams.

HARROGATE Infirmary has received £60 from the Knaresborough Amalgamated Friendly Societies, which was a record collection.

THE Right Rev. Handley Carr Glyn Moule, Bishop of Durham, has left £300 for a memorial bed at Nablow's Hospital, Palestine.

MR. F. W. LAWRENCE, of Bath, has left £100 each to the Royal United Hospital and the Royal Mineral Water Hospital and £50 to the Eye Infirmary.

CAROLINE INEZ COUNTESS OF CAVAN, wife of the Earl of Cavan, who commanded the 10th Army in the late war, left £100 to the Welwyn Cottage Hospital.

THE residue of the property of Mr. C. E. Noverre, Chairman of the London Board of the Norwich Union Life Office, has been left to the Jenny Lind Infirmary for Sick Children, Norwich.

THE Royal Victoria Hospital, Belfast, receives a gift of £1,000 from San Francisco, being a sum given by Mrs. Wallace, the daughter of a Belfast man. This donation is to enable the work of the Throne Convalescent Hospital for Children to be extended.

THE North Staffordshire Cripples' Aid Society has received a sum of £124 1s. 10d. from the executors of the late Thomas Tunstall, which was subject to the life interest of the widow. This is in addition to £456 15s. 1d. paid on the death of the deceased in December 1916. This Society has also received from the trustees of the Hanchurch Convalescent Home (which has been handed over to the Stoke-on-Trent Corporation) a sum of £69 12s. 2d. and £839 1s. 4d. India 3 per cent. stock, representing the balance after winding up.

